# The fate of the neuromuscular junction in peripheral nerve injury – current understanding and future research aims: A scoping review

**DOI:** 10.1016/j.jpra.2026.04.016

**Published:** 2026-05-01

**Authors:** Joseph Overland, Alexander Savage, Nicholas Savage, Camille LaLiberte, Aaron Dingle, Lipi Shukla

**Affiliations:** a Plastic, Reconstructive, and Hand Surgery Unit, St Vincent’s Hospital Melbourne, 41 Victoria Parade, Fitzroy, VIC 3065, Australia; b Plastic, Hand & Faciomaxillary Surgery Unit, Alfred Health, 55 Commercial Road, Melbourne, VIC 3004, Australia; c Division of Plastic & Reconstructive Surgery, Department of Surgery, School of Medicine and Public Health, University of Wisconsin-Madison, G5/361 Clinical Science Center 600 Highland Avenue, Madison, WI, 53792-7375, USA

**Keywords:** Peripheral nerve injury, Neuromuscular junction, Motor endplate, Reinnervation, Nerve regeneration, Nerve repair

## Abstract

**Importance:**

Peripheral nerve injury (PNI) represents a challenging frontier for the many surgical specialties dealing with these injuries. Improved surgical techniques and understanding have resulted in improvements in patient care, but a mastery of the processes of nerve regeneration and repair have proved elusive.

**Objective:**

This review intends to outline the changes at the neuromuscular junction in response to denervation injury. By reviewing the current literature, we aim to solidify the understanding of these morphological changes and identify future areas of research need.

**Review:**

A literature search of PubMed, Embase and Web of Science including studies up to 22nd December 2023 was carried out. Search terms related to the motor endplate or neuromuscular junction, nerve degeneration, peripheral nerve injury and nerve regeneration. Three researchers independently performed screening of potentially relevant studies, using *Covidence* (Melbourne, Australia), a review workflow platform facilitating blinded reviewing. Results are reported in accordance with the Preferred Items for Systematic Reviews and Meta-analysis (PRISMA) guidelines

**Findings:**

We identified 3595 records in the initial search, with no additional articles identified through backward and forward citation tracking. 350 full-text reports were assessed for eligibility. Ultimately, 118 studies were included for data extraction.

**Conclusions and Relevance:**

The mechanisms of degeneration and regeneration at the NMJ summarized within provide multiple opportunities for ongoing research. Terminal Schwann cells and the constituents of the post synaptic apparatus may be able to be induced to survive for longer, or to be replenished to levels facilitating functional recovery from greater periods of denervation.

**Key points**

 

- Terminal Schwann cell (TSC) staining correlates almost precisely with acetylcholine receptor staining in the uninjured neuromuscular junction (NMJ).
- The NMJ can have different characteristics depending on the muscle fibre type. They can switch type when innervated by a different neuron.
- There is limited correlation between change in NMJ morphology in response to injury and function.
- Acetylcholine receptors are in some models dependent on connection with the terminal axon for survival. This is also true of TSCs.
- Blurring/fragmentation of the acetylcholine receptors may represent the “point of no return” for functional reinnervation. Whether from resection, transection or chronic repetitive denervation, reinnervation of the NMJ does not seem possible beyond this point.
- Nerve research is challenging due to contradictory findings/lack of ideal model. Animal models demonstrate distinct models of injury – and even within the same animal model differences in time points since injury add to these differences. Conceptually, a molecular marker of injury and/or regeneration could be useful in both research and patient treatment.
- While reinnervation of the NMJ does not prevent fragmentation completely, branching from other terminal axons does compensate.
- Neurite branching is replaced by proximal growth through the myelin sheath – it is not always the original fibre that replaces the sprouted fibre. This is yet another reason for decreased function post nerve injury and a potential target for research.
- Polyinnervation – whereby a motor endplate is innervated by a sprouted neurite and a proximal regenerated neuron – is a feature of partial nerve injury.
- A single axon can sprout several times and innervate multiple MEPs. This leads to an overall increase in the muscle function per motor unit, but is limited to approximately 4 MEPs per single axon.
- In early reinnervation, the majority occurs via terminal sprouting (endplate to endplate), but later changes to a mix of terminal and collateral sprouting.

Alt-text: Unlabelled box dummy alt text

## Introduction

Peripheral nerve injury (PNI) represents a challenging frontier for the patients and many surgical specialties dealing with these injuries. Despite the range of surgical options, the time required for reinnervation remains crucial in outcomes following repair. Nerve regeneration is a complex physiological process involving multiple steps, cells and sites, any of which may unlock faster and more effective recovery.

The neuromuscular junction (NMJ) is a crucial target along the regenerative pathway. Basic teaching dictates that dennervated motor end plates (MEPs) have a limited window for successful reinnervation. A critical threshold is reached whereby axonal contact with the motor endplate fails to provide functional recovery. The processes at the NMJ and the time critical nature of establishing re-innervation remain poorly understood. The authors provide an overview of the existing literature on these processes, aiming to enhance understanding, identify knowledge gaps, and guide future research in this field.

## Method

A literature search of PubMed, Embase and Web of Science including studies up to 22nd December 2023 was carried out. Search terms were developed with the institution’s research librarian. Terms related to the motor endplate or neuromuscular junction, nerve degeneration, peripheral nerve injury and nerve regeneration. The complete search strategy is available within the appendix. Additional articles were identified through citation tracking of published systematic reviews and included studies.

Identified titles and abstracts were screened for inclusion based on the following criteria: publications were written in English and described endplate or neuromuscular junction morphology in the setting of peripheral nerve injury through cut or crush. Injury through freeze, toxin-mediated or other mechanisms were excluded. Studies including genetic knock-out animal models without a control were also excluded. The primary outcome was neuromuscular junction morphology following denervation injury.

Three researchers (J.O., N.S. and A.S.) independently performed title and abstract screening as well as full-text review, using *Covidence* (Melbourne, Australia). Disagreements prompted discussion and were resolved by consensus. Three reviewers (J.O., N.S. and A.S.) independently extracted study data using a prespecified data abstraction form. Quality of included studies was assessed as part of inclusion criteria, which pertained to histomorphological examination of the endplate in the instance of nerve injury. Quality assessment tools for in vivo experimental studies are not currently well validated and were not formally employed.

Results are reported in accordance with the Preferred Items for Systematic Reviews and Meta-analysis (PRISMA) guidelines. This study does not require institutional ethics review, although the protocol was registered prospectively with PROSPERO (CRD42024495976). Quantitative analysis was accomplished with the use of Microsoft Excel, version 16.87 (Microsoft Corporation).

## Results

**Figure 1:**
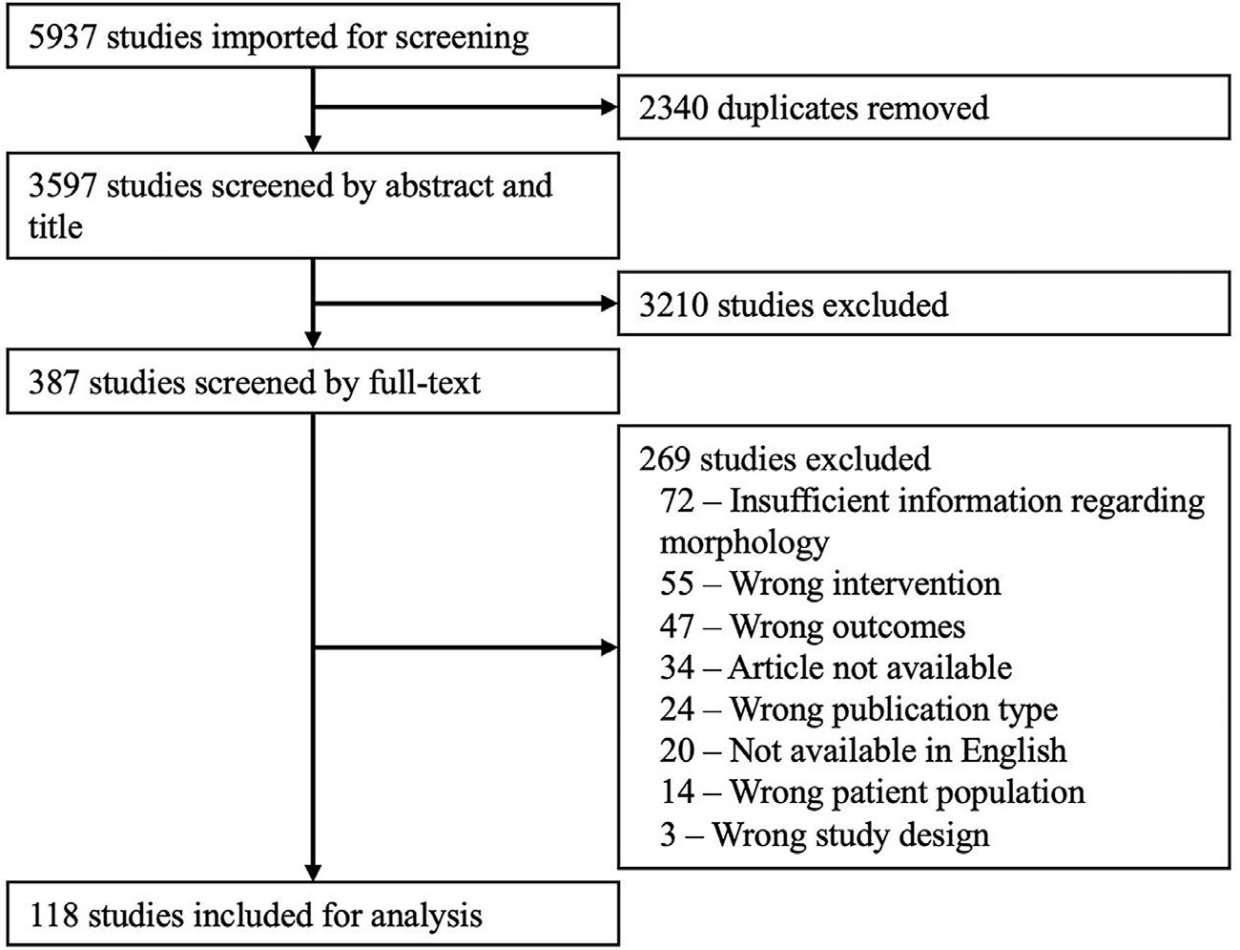
Review flowchart.

**Figure 2:**
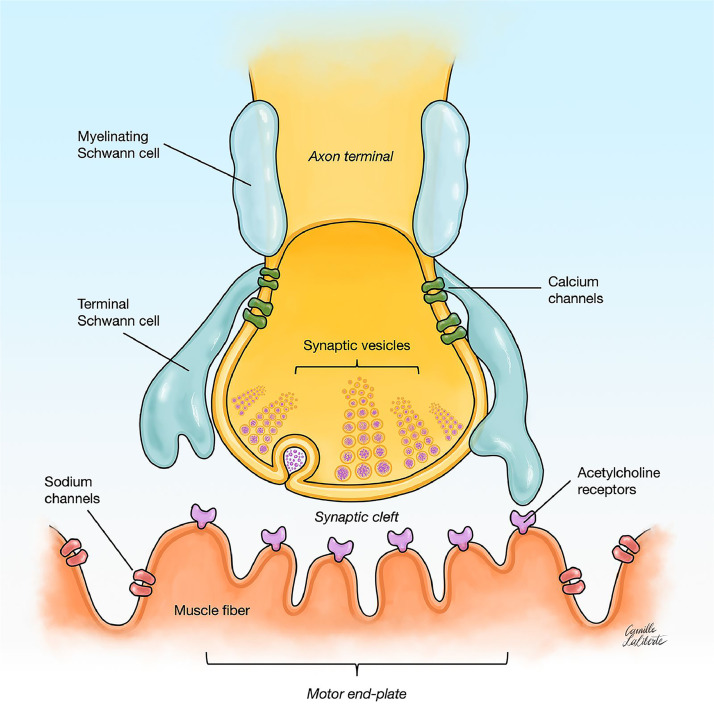
The neuromuscular junction.

**Figure 3:**
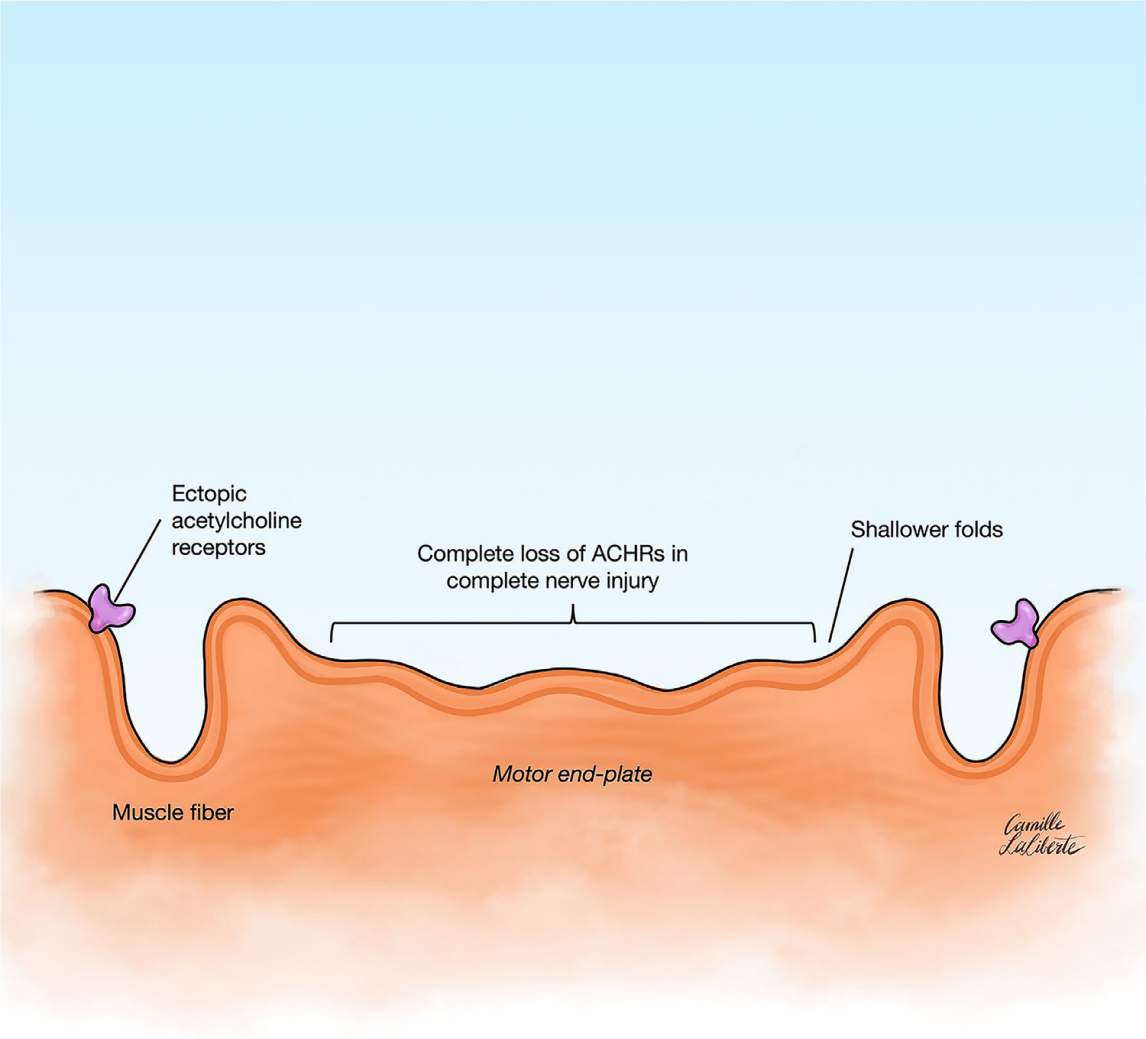
Post-synaptic changes after denervation.

**Figure 4:**
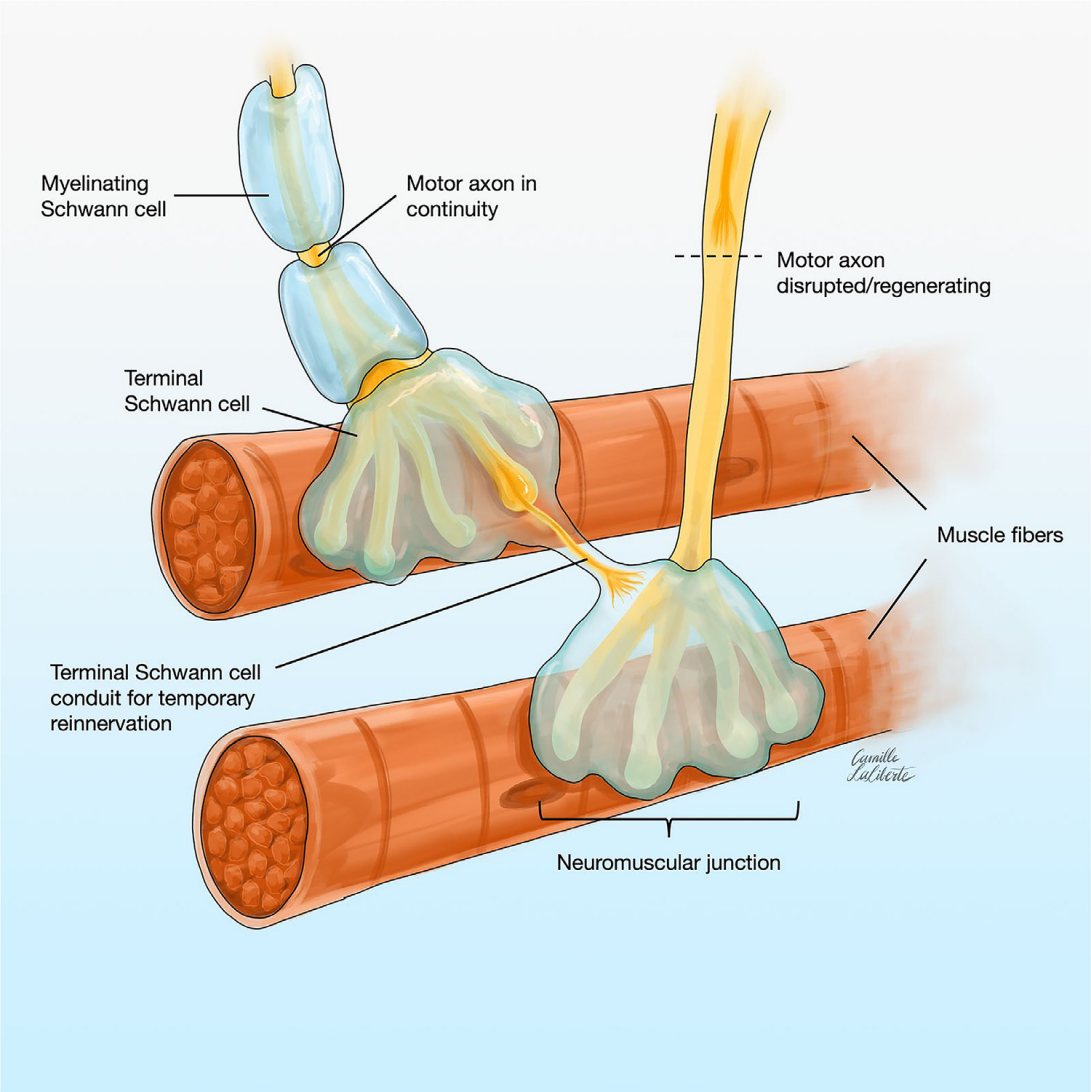
Terminal Schwann cell sprouting in partial denervation.

**Figure 5:**
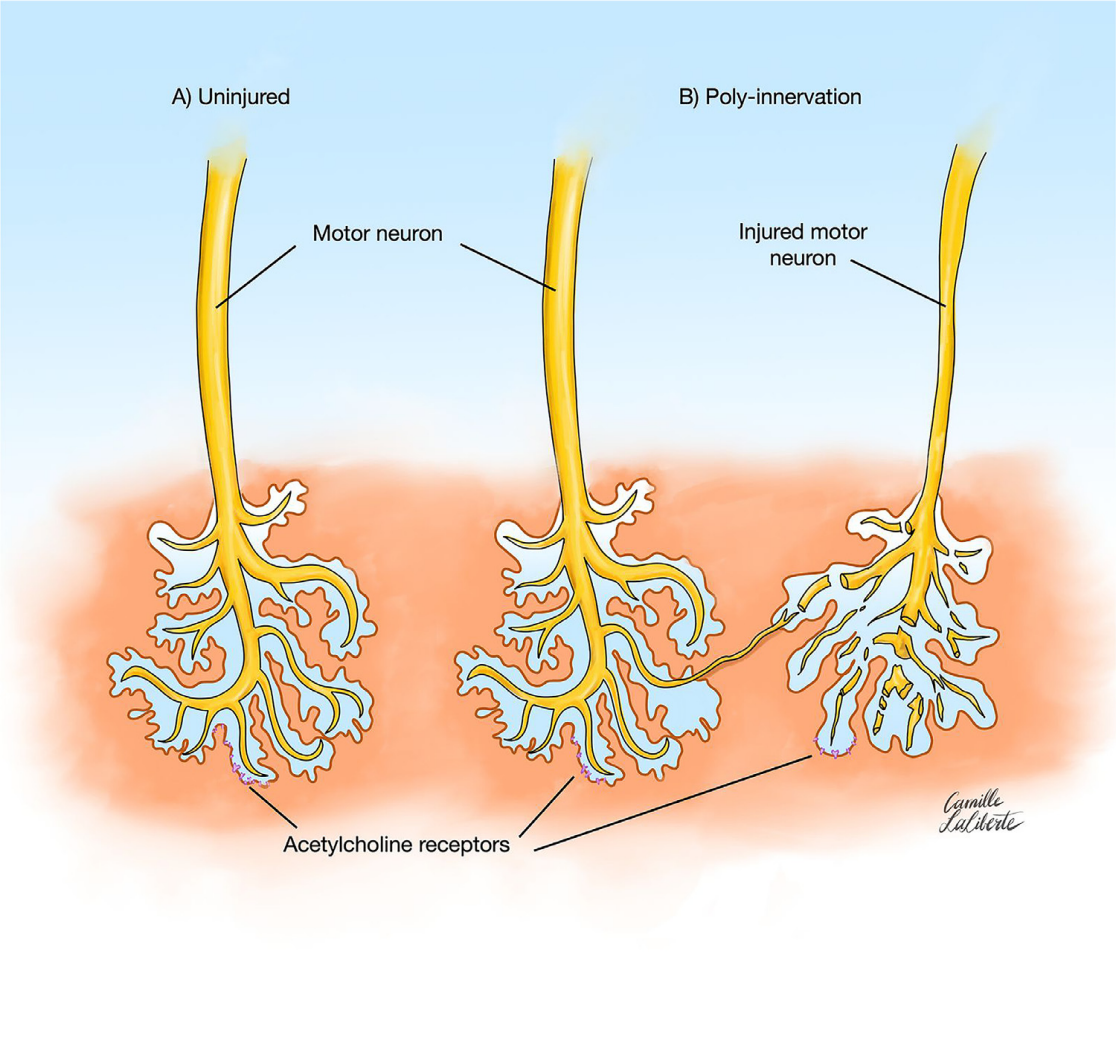
Polyinnervation of motor endplates.

We identified 3595 records in the initial search, with no additional articles identified through backward and forward citation tracking. In total, 350 full-text reports were assessed for eligibility. The review flowchart study selection is shown in Figure 1. Ultimately, 118 studies were included for data extraction (Figure 2, Figure 3, Figure 4, Figure 5).

Table 1 shows the breakdown of the studies included (article type, animal type, injury type, injury location, morphology assessment). Table 2 shows those studies in which an intervention was studied and details the intervention.

**Table 1:** Pre-clinical studies.

Type of animal	Author	Year	Sample size	Type of injury	Location of injury	Treatment present	Follow-up length (d)	Assessment type*
Rat	Eranko et al.	1967	30	Cut	Sciatic n.	No	240	C
	Miledi et al.	1970	Unclear	Cut	Other peripheral n.	No	Not stated	D
	Gonzenbach et al.	1973	Unclear	Cut	Phrenic n.	Synthetic nerve graft	270	D, G
	McIsaac et al.	1975	84	Crush	Sciatic n.	No	35	C, E
	Frank et al.	1976	Unclear	Cut	Sciatic n.	Electrical stimulation	120	A
	Wærhaug et al	1977	Unclear	Cut	Tibial n. or peroneal n.	Coaptation	270	G
	Strand et al.	1980	Unclear	Crush	Sciatic n.	No	56	C, G
	Hatano	1981	60	Cut	Sciatic n.	Coaptation	213	C
	Gorio et al.	1983	Unclear	Crush	Sciatic n.	No	35	C, D
	Eberstein et al.	1986	Unclear	Crush	Tibial n. or peroneal n.	Electrical stimulation	18	D, G
	Jay et al.	1989	Unclear	Cut	Sciatic n.	No	730	A, C
	Pachter et al	1989	18	Crush	Tibial n. or peroneal n.	No	11	D
	Pachter et al.	1991	Unclear	Cut	Lumbosacral plexus root	No	30	C, E
	Ribaric et al.	1991	55	Crush	Sciatic n.	No	35	D
	Reynolds et al.	1992	38	Both	Sciatic n.	No	60	A, B
	Woolf et al.	1992	Unclear	Both	Sciatic n.	No	60	B, C
	Bruno et al.	1993	Unclear	Crush	Sciatic n.	No	60	C
	Mehta et al.	1993	16	Both	Sciatic n.	No	14	B, C, E, G
	Ann et al.	1994	Unclear	Crush	Other peripheral n.	No	30	D, 8
	Parson et al.	1997	Unclear	Cut	Sciatic n.	No	7	C, E
	Love et al.	1998	Unclear	Both	Other peripheral n.	No	8	B
	Ribera et al.	1998	4	Cut	Sciatic n.	No	30	D, B
	Shiotani et al.	1998	36	Cut	Other cranial n. or branch	No	28	C, E
	Flint et al.	1999	22	Cut	Other cranial n. or branch	Other	30	C
	Love et al.	1999	Unclear	Both	Other peripheral n.	No	12	B
	Shiotani et al.	1999	48	Cut	Other cranial n. or branch	No	28	C, E
	Becker et al.	2000	66	Cut	Tibial n. or peroneal n.	Coaptation	66	B, D
	Simon et al.	2000	Unclear	Cut	Sciatic n.	No	120	A
	Dekkers et al.	2001	Unclear	Crush	Sciatic n.		21	B
	Ijkema-Paassen et al.	2001	16	Cut	Tibial n. or peroneal n.	Other	147	C
	Ijkema-Paassen et al.	2002	11	Cut	Sciatic n.	Other	147	C
	Papkonstantinou et al.	2002	10	Cut	Brachial plexus other including peripheral branches	Coaptation	210	C
	Rodella et al.	2002	70	Cut	Sciatic n.	No	60	C
	Zhou et al.	2002	46	Crush	Sciatic n.	No	140	A, B
	Love et al.	2003	Unclear	Cut	Other peripheral n.	No	7	A, B
	Tam et al.	2003	43	Cut	Lumbosacral plexus root	No	30	B, C, E
	Maeda et al.	2004	42	Cut	Hypoglossal n. or branch	No	35	A, B, D
	Wang et al.	2004	50	Crush	Sciatic n.	No	140	B
	Guntinas-Lichius et al.	2005	72	Cut	Facial n. or branch	Other	56	B
	Keilhoff	2005	60	Cut	Other lumbosacral plexus branch	No	56	B, C, D
	Kumai et al.	2005	48	Cut	Other cranial n. or branch	No	70	A, B
	Evgenieva et al.	2008	78	Cut	Hypoglossal n. or branch	Coaptation	60	A
	Mozaffar et al.	2008	Unclear	Both	Sciatic n.	No	56	A, B
	Lain et al.	2009	Unclear	Both	Sciatic n.	Transfer	10	B
	Favero et al.	2010	Unclear	Crush	Other peripheral n.	Electrical stimulation	22	A
	Gu et al.	2010	75	Cut	Sciatic n.	Other	213	B
	Magill et al.	2010	8	Crush	Facial n. or branch	No	56	A
	Pranaite et al.	2010	44	Cut	Sciatic n.	Synthetic nerve graft	98	A
	Grumbles et al.	2012	Unclear	Cut	Sciatic n.	No	46	A, B
	Jiang et al	2012	Unclear	Cut	Tibial n. or peroneal n.	Other	84	D
	Jonsson et al.	2013	19	Cut	Sciatic n.	Coaptation	91	A, B
	Akdeniz et al.	2015	30	Cut	Sciatic n.	Coaptation	56	C, E
	Willand et al	2015	29	Cut	Tibial n. or peroneal n.	Coaptation	90	A
	Sakuma et al.	2016	Unclear	Both	Sciatic n.	Coaptation	38	D, B
	Ruven et al.	2017	Unclear	Cut	Brachial plexus other including peripheral branches	No	84	A, G
	Li et al.	2020	36	Cut	Brachial plexus root	No	35	B
	Lukacova et al.	2020	50	Crush	Other peripheral n.	No	8	B
	Pinto et al.	2020	40	Cut	Sciatic n.	Coaptation	60	A, B
	Ito et al.	2022	18	Cut	Tibial n. or peroneal n.	Other	28	A, B
	Walter et al.	2022	Unclear	Cut	Sciatic n.	No	14	A
	Tiburcio et al.	2023	40	Cut	Sciatic n.	Coaptation	30	B, G
Mouse	Saito et al.	1969	Unclear	Cut	Sciatic n.	No	266	C, D, E
	Brown et al.	1978	Unclear	Both	Lumbosacral plexus root	No	26	C
	Brown et al.	1982	8	Cut	Sciatic n.	No	15	C
	Slack et al.	1982	Unclear	Both	Lumbosacral plexus root	No	400	C, D, E
	Rochel et al.	1988	Unclear	Cut	Lumbosacral plexus root	No	90	G
	Rich et al.	1989	204	Both	Brachial plexus other including peripheral branches	No	38	A, C, E
	Shyng et al.	1990	Unclear	Both	Other cranial n. or branch	No	18	A
	Bewick et al.	1991	21	Cut	Sciatic n.	Coaptation	180	E
	O'Malley et al.	1999	Unclear	Cut	Brachial plexus other including peripheral branches	No	30	A, B
	Szabo et al.	2003	Unclear	Crush	Brachial plexus other including peripheral branches	No	Not stated	A, D
	Gillingwater et al.	2004	Unclear	Crush	Tibial n. or peroneal n.	No	42	A
	Marques et al.	2006	40	Crush	Other cranial n. or branch	No	14	A, B
	Magill et al.	2007	10	Crush	Sciatic n.	No	42	A
	Thams et al.	2009	Unclear	Both	Sciatic n.	No	45	A
	Ma et al.	2011	Unclear	Cut	Sciatic n.	Coaptation	55	B
	Murray et al.	2011	Unclear	Both	Tibial n. or peroneal n.	No	Not stated	A
	Seitz et al.	2011	48	Cut	Facial n. or branch	Coaptation	Not stated	A, B
	Kang et al.	2014	33	Crush	Other cranial n. or branch	No	10	A, B
	Lavasani et al.	2014	Unclear	Cut	Sciatic n.	Other	126	B
	Macpherson et al.	2015	Unclear	Cut	Tibial n. or peroneal n.	Transfer	42	A, B
	Dalkin et al.	2016	Unclear	Crush	Tibial n. or peroneal n.	No	4	B
	Zainul et al.	2018	Unclear	Crush	Sciatic n.	No	42	B
	Kang et al.	2019	Unclear	Crush	Other cranial n. or branch	No	10	A, B
	Vannucci et al.	2019	31	Cut	Sciatic n.	Coaptation	210	A, B
	Li et al.	2020	130	Both	Tibial n. or peroneal n.	Other	112	A, B
	Lu et al.	2020	Unclear	Cut	Sciatic n.	Coaptation	42	A, B
	Ojeda et al.	2020	Unclear	Both	Facial n. or branch	No	Not stated	A, B
	Brayman et al.	2021	Unclear	Crush	Tibial n. or peroneal n.	No	Not stated	B
	Bermedo-Garcia et al.	2022	Unclear	Crush	Facial n. or branch	No		B, D
	Li et al.	2022	160	Cut	Tibial n. or peroneal n.	Nerve graft	112	A, B
	Perez-Gonzalez et al.	2022	Unclear	Both	Sciatic n.	No	18	A, B
	Jablonka-Shariff et al.	2023	81	Cut	Tibial n. or peroneal n.	Fibrin glue	42	A, B
	Kosco et al.	2023	Unclear	Crush	Sciatic n.	No	28	B
	Lu et al.	2023	69	Cut	Sciatic n.	Coaptation	56	A, G
Frog	Dennis et al.	1974	Unclear	Crush	Brachial plexus other including peripheral branches	No	21	C
	Letinsky et al.	1976	Unclear	Crush	Brachial plexus other including peripheral branches	No	90	C
	Verma et al.	1977	Unclear	Cut	Sciatic n.	No	Not stated	D
	Decino	1981	Unclear	Crush	Brachial plexus other including peripheral branches	Other	30	C
	Ding	1982	Unclear	Crush	Brachial plexus other including peripheral branches	No	280	C
	Morrison-Graham et al.	1983	Unclear	Both	Brachial plexus other including peripheral branches	No	10	C, D
	Herrera et al.	1985	54	Crush	Other peripheral n.	Unclear	517	C
	Krause et al.	1985	Unclear	Cut	Brachial plexus other including peripheral branches	Unclear	750	A, C
	Werle et al.	1991	Unclear	Crush	Other peripheral n.	No	730	C
	Stanco et al.	1997	18	Both	Branchial plexus other including peripheral branches	No	30	A, B
	Astrow et al.	1998	Unclear	Cut	Branchial plexus other including peripheral branches	No	28	A, B
	Stanco et al.	1998	Unclear	Unclear	Sciatic n.	No	Not stated	B
	Koirala et al.	2000	6	Cut	Other peripheral n.	No	70	B
Cat	Carlsson et al.	1971	Unclear	Cut	Lumbosacral plexus root	No	266	C, F, G
	Desantis et al.	1979	Unclear	Cut	Other peripheral n.	No	7	D
Rabbit	Richter et al.	1982	75	Cut	Tibial n. or peroneal n.	Coaptation	365	D, E, F
Monkey	Lin et al.	2012	24	Cut	Lumbosacral plexus root	No	56	D
Other	Atwood et al.	1974	Unclear	Both	Other peripheral n.	No	340	D
	Matsuda et al.	1988	Unclear	Cut	Sciatic n.	Coaptation	140	D

⁎ Key for assessment type: A = Alpha Bungarotoxin (AChR) staining. B = Immunofluorescence. C = Acetylcholinesterase staining. D = Electron microscopy. E = Silver Staining. F = Methylene Blue. G = Other.

**Table 2:** Observational studies in humans.

Article type	Author	Year	Sample size	Type of injury	Location of injury	Treatment present	Follow-up length (d)	Assessment type^a^
Prospective cohort	Chan et al.	2019	2	Unclear	Brachial plexus other including peripheral branches	No	150	A
Prospective cohort	Gupta et al.	2020	18	Unclear	Brachial plexus other including peripheral branches	No	2190	A, B
Prospective cohort	Chen et al.	2023	1	Unclear	Brachial plexus other including peripheral branches	No	98	B
Retrospective cohort	Gupta et al.	2023	10	Unclear	Brachial plexus other including peripheral branches	No	4957	B

a Key for assessment type: A = Alpha Bungarotoxin (AChR) staining. B = Immunofluorescence. C = Acetylcholinesterase staining. D = Electron microscopy. E = Silver Staining. F = Methylene Blue. G = Other.

## Discussion

### The uninjured neuromuscular junction

The NMJ comprises the terminal axon, which is covered by the terminal Schwann cell (TSC) and contacts the muscle cell at the MEP.^1^ These structures are located along characteristic bands of each muscle.^2^ Structural changes in neurodegenerative diseases suggest that NMJ structure and function are closely linked.^3^

The axon terminal is bulb-shaped,^4^ containing numerous synaptic vesicles and mitochondria.^5^ The myelin sheath of the terminal axon stops just short of the NMJ; here the axon is covered by a bell-shaped TSC, which plays an important role in NMJ homeostasis.^6^ TSC staining correlates closely to acetylcholine receptors (AChR) staining in uninjured specimens^7^ – 9% of TSCs processes narrowly extend past the AChR area.^8^

Upon exiting the sheath, the terminal axon divides into branches sitting within the synaptic cleft, separated from muscle by a layer of basement membrane.^9^ The primary synaptic cleft lies between axon and a cup-shaped depression on the muscle surface,^10^ with the secondary synaptic gap lying between the folds of muscle cell membrane.^5^ These folds greatly increase the surface area of the endplate.^11^ There is typically one axon fibre per MEP.^12^

At the MEP periphery are pretzel-shaped aggregations of AChRs.^13^^,^^14^ These are completely covered by the terminal, perforated and well-defined.^15^ Mature MEP morphology is “pretzel-like”, compared to plaque-like during development and in immature MEPs.^16^ Acetylcholinesterase (AChE) is also present at the NMJ; found in the periphery of synaptic folds, less intensely in the centre of the terminal axon and not in muscle or TSCs.^17^

NMJs display different characteristics depending on the muscle fibre type they innervate and can change when reinnervated by a different motor neuron.^18^

## The injured state

### Post-synaptic apparatus

The post-synaptic apparatus, consisting of the MEP and associated acetylcholine receptors, is initially more resilient to PNI than is the pre-synaptic apparatus.^7^^,^^19^^,^^20^ In rats, AChRs can persist for months following injury.^20^ The morphology of the injured MEP is established, moving from “pretzel” to “plaque”-like appearance.^21^ PNI causes the MEP to degenerate to this earlier developmental plaque-like state.^22^

#### The endplate

Immediately after injury, the AChE staining becomes weaker, diminishing further over the first month following injury.^17^ Total MEP number may increase but the morphological changes are significant. Notable early changes include a decrease in size,^14^^,^^23^, ^24^, ^25^, ^26^, ^27^ fragmentation and heterogenous staining,^14^^,^^23^^,^^27^, ^28^, ^29^, ^30^ fewer and more shallow folds^9^^,^^31^ and decreased density.^32^ Reinnervation can reverse these changes to mature morphology^14^^,^^27^^,^^33^^,^^34^ and pre-injury staining levels.^17^^,^^33^ Fragmentation can also persist despite reinnervation.^30^ In the absence of reinnervation, the staining fades to become virtually undetectable.^17^^,^^27^^,^^34^ The sub-neural apparatus shrinks laterally and becomes elongated.^17^

Denervated human MEPs are plaque-like; appearing more fragmented, with faded synaptic gutters and asymmetrical AChR staining.^22^ Biopsied human muscle 14 weeks post non-transecting injury to axillary nerve showed MEP regression from compact pretzels to more dispersed and enlarged intermediate morphologies, although some pretzel MEPs persisted.^16^ Remnant morphologically normal MEPs have been observed beyond 3 years in brachial plexus injuries.^22^ This study found human denervated MEPs decrease in volume and surface area between 0 and 10 months, but these parameters normalised between 1.5 and 3 years after denervation in patients who underwent surgical intervention due to lack of clinical recovery. Even though the authors state this was in comparison to ‘normal’ muscle, this data was not presented therefore limiting further comparisons.^22^ Morphological changes to the MEP in humans do not appear to be muscle specific.^22^

Analysing both rats and mice, Sakuma et al.^35^ found that at 4 months post-transection and re-suture, the post-synaptic apparatus of the NMJ showed accumulations of mitochondria in the transection/multiple crush group. This feature is sometimes seen in neurodegenerative conditions.^36^

#### Acetylcholine receptors

AChRs are embedded within the basement membrane of the endplate.^37^ AChRs are eventually lost following denervation/reinnervation injury, with increased time associated with greater loss of post-synaptic receptors.^38^ Kang et al. showed that increasing denervation period from 4 to 9 days post crush injury resulted in a greater NMJs receptor loss, of >5%, in a murine model.^38^ Receptor loss is more significant when the nerve is severed and then closely opposed allowing prolonged reinnervation.^38^^,^^39^ The loss of AChRs may be directly proportional to the amount of polyinnervation of endplates (discussed later).^39^

Gain of receptors has also been noted post-denervation/reinnervation.^38^ Ectopic receptor formation in the extra-junctional portion of the muscle occurs post crush injury but, disappear with reinnervation.^13^ Ectopic receptors are also prominent following denervation^40^ but are absent in uninjured NMJs.^20^

Some AChR plaques show signs of de-differentiation following denervation and reinnervation – becoming smaller with less intense staining, less perforations, flattening, absence of branching and fragmentation^7^^,^^15^^,^^38^ AChR sites have been found to initially shrink immediately post crush injury, but this improves with reinnervation, sometimes to near normal, at 12 weeks and 90 days in two separate studies.^7^^,^^13^ This shrinkage has also been observed in human MEPs.^41^

With irreversible denervation, AChR sites move from pretzel-like to fragmented and blurry by day 30,^13^ and the proportion of ectopic receptors increases.^13^ It is postulated that post-synaptic fragmentation takes two forms – fragmented smooth and fragmented blurred.^13^ Fragmented smooth AChR clusters are associated with nerve crush and subsequent reinnervation whereas fragmented blurred post synaptic AChRs clusters are unstable and are associated with chronic denervation/nerve resection.^13^ This blurred fragmentation potentially marks the point where complete functional reinnervation is no longer possible.

Early loss of AChRs after denervation appears dependent on partial reinnervation. When reinnervation is prevented, many studies show no associated early loss of AChRs^13^^,^^38^^,^^39^ However, other studies have suggested the loss of AChR area can occur acutely, even when reinnervation is prevented.^19^ AChRs may be lost from those areas innervated by sprouting axons which regress in the face of reinnervation of the same MEP by a sprout from the endoneurial tube i.e. polyinnervation.^38^

Chronic denervation with prevention of reinnervation results in loss of receptors. In an amphibian model of NMJ, those denervated for >500 days without reinnervation showed no binding of alpha-bungarotoxin at the MEP, but did find evidence of ectopic receptors.^42^ This suggests a dependency on the terminal axon for survival. Other studies show in denervated NMJs observed beyond 6 weeks, AChRs are lost.^32^

Some of the challenges inherent to PNI research are outlined in the findings above, which at times can seem contradictory. Animal models demonstrate distinct responses, even within the same muscle.^43^ Different times points also yield distinct results, while only providing a static picture – dynamic assessment is not currently possible. Rather than relying on histological snap shots, clinical decision making could be augmented by a molecular marker of recovery of the neuromuscular junction. This has been explored by positron emission tomography through glutamate carboxypeptidase II ligands, demonstrating possible use as a biomarker of muscle denervation.^44^

#### Terminal Schwann cells

TSCs remain attached to the synaptic site immediately after denervation,^45^^,^^46^ covering the AChR in the muscle fibre membrane.^38^ Without innervation, they are only transiently maintained.^2^^,^^9^^,^^45^ In mice, 72% of NMJs show some loss of TSC coverage at 4 days post nerve injury, increasing to 100% by day 10. The extent of lost coverage increases from 13% at day 4 to 28% at day 10.^38^ Without reinnervation, by day 28, 80% of mice TSCs are not visible at the NMJ, occurring more quickly in frogs.^47^^,^^48^ Reinnervation can reverse some of these changes.^13^^,^^49^

In partial denervation, TSCs from innervated MEPs will sprout^8^ and make connections with denervated MEPs.^11^^,^^12^^,^^50^^,^^51^ Axonal regrowth will occur via these bridges.^12^^,^^50^^,^^51^ A stimulatory role of reinnervation in the setting of partial denervation is supported by: a) less frequent bridging seen in completely denervated muscle, b) increased bridging frequency as synaptic function returns to partially denervated muscle, and c) by crush causing partial denervation coupled with botulinum toxin decreasing bridge formation five-fold.^50^^,^^51^ Growth-associated phosphoprotein 43 (GAP 43)^52^^,^^53^ and vascular endothelial growth factor (VEGF) signalling^54^ have been implicated in bridge formation.

TSCs migrate toward denervated NMJs in response to injury,^13^^,^^50^ initially increasing TSC number near the NMJ.^8^ This decreases to at least baseline TSC number in more chronic injuries.^13^^,^^55^

TSCs are phagocytic,^49^ displaying phagocytosis markers in response to injury.^56^ The sensitivity of their two main receptors – muscarinic and purinergic – may be involved in switching phenotypes from maintenance to repair. Decreased muscarinic sensitivity is seen in all PNI, whilst purinergic activity is unchanged by denervation but decreased during reinnervation^56^ Decreased muscarinic receptors are observed well after recovery – suggesting “glial memory” post injury.^56^ TSCs also contribute to MEP maintenance post injury by releasing ACh.^57^ They play an important role in guiding the terminal axon branching post denervation injury,^8^^,^^45^ and their ablation at the time of injury leads to decreased reinnervation.^6^ After crush injury in rats, TSCs recover more quickly than the nerve terminals and AChRs of the same NMJ – indicating a potential role in guiding regeneration.^7^

#### Regenerating axons into the NMJ

Axons fragment within days of injury.^9^^,^^30^^,^^40^ The regenerating axons are initially very thin,^4^ forming new junctions at previous sites rather than de-novo.^39^^,^^49^^,^^58^, ^59^, ^60^ In partial denervation, these reconnections are initially formed via ultra-terminal sprouting^11^^,^^61^^,^^62^ and collateral sprouting^62^ which grow along pre-existing TSC processes and cell bodies.^45^^,^^51^^,^^63^ A single axon can sprout several times and innervate multiple MEPs.^12^ This leads to an overall increase in the muscle function per motor unit,^12^ but is limited to approximately 4 MEPs per axon.^64^ In early reinnervation, the majority occurs via terminal sprouting (endplate to endplate), but later changes to a mix of terminal and collateral sprouting.^65^ While reinnervation does not prevent MEP fragmentation, branching of the terminal axon does appear to compensate to a degree.^30^

Neurite regrowth is inhibited by synapse reformation, but proximal branching allows innervation of more distal terminals.^46^ Later, reinnervation of proximal nerve fibres from the growth cone can displace sprouting fibres, although it is not always the original nerve fibre which replaces the sprouted fibre.^62^ Regeneration of injured axon is fast in the nerve trunk, slowing significantly in the synaptic cleft, potentially explaining the lag between synapse connection reformation and functional recovery.^46^

Polyinnervation of MEPs is the rule in reinnervation.^2^^,^^4^^,^^7^^,^^9^^,^^13^^,^^14^^,^^27^^,^^49^ It usually arises from separate axon;s^66^ typically a terminal sprout from an adjacent innervated NMJ, and an axon from the original Schwann cell tube.^39^ While synaptic function appears worse in polyinnervation compared to monoinnervation,^67^ competing terminals at the same NMJ are both functional during reinnervation.^56^ Fibroblast growth factor 2 (FGF2) may be protective, with increased rates of polyinnervation in FGF2 knockout mice following nerve injury.^68^

Polyinnervation also persists following established reinnervation,^13^ but does resolve eventually via spontaneous regression.^46^ This transition from polyinnervation to monoinnervation post-crush is conditional on contact with the motor neuron cell - it does not occur with induced nerve block or by electrical stimulation distal to the level of injury.^69^ Regression of polyinnervation can be inhibited by glial cell line-derived growth factor, but at the expense of functional reliability and neuronal integrity.^70^

### Future directions for NMJ optimisation

Future enhancements of motor function outcomes for our patients after peripheral nerve injury will require optimisation of both axonal regeneration and motor end plate preservation. Despite this, many immerging therapeutic interventions for PNI such as growth factors,^71^ electrical stimulation^72^ and postoperative exercise regimes^73^ largely focus on improving axonal regeneration. It is important to ask what effect do these interventions have at the NMJ and what other interventions may specifically target this locale along the regenerative pathway of PNI? The low number of human NMJ studies (Table 2) identified by our review reflects the challenges faced by peripheral nerve scientists. These challenges include uneven distribution of NMJs in humans making accurate biopsy more difficult, ethical considerations regarding serial biopsy and biopsy of uninjured muscles for control, and timing mismatch between clinical interventions and NMJ morphological changes. Thus, many of the future directions are hypothesized from animal models.

#### Stem cells

Stem cells represent a possible frontier for peripheral nerve surgery. Many pre-clinical studies have assessed intervention at direct nerve coaptation,^74^^,^^75^ autografts^76^^,^^77^ and allograft,^78^^,^^79^ as well as stem cell embedded nerve conduits.^80^ Specific findings at the NMJ following stem cell application are sparse but have shown that during regeneration post nerve repair, injection of spinal cord stem cells distal to the site of ligation followed by release 6 weeks later, result in a greater percentage of morphologically normal and larger endplates.^23^ Motor neurons from injected spinal stem cells can reinnervate NMJs, forming immature plaque-like structures.^81^ Injection of foetal neural stem cells into the distal stump of a resected nerve show some reinnervation in a rat model.^82^ Injection of human muscle derived stem cells into the site of critical mouse sciatic nerve gap resulted in return to near-normal MEP morphology, compared to sham injections.^83^

#### Electrical stimulation

Electrical stimulation has been investigated in peripheral nerve injury as early as the 1950s^84^ and has been studied extensively in pre-clinical and clinical models.^72^ Electrical stimulation of muscle postoperatively in the setting of surgical denervation significantly decreases both extra-junctional and junctional AChRs.^20^ Electrical stimulation distal to site of nerve injury does not decrease polyinnervated NMJs^69^ but does lessen the flattening of the synaptic clefts.^10^

Electrical stimulation decreases the atrophy which accompanies denervation.^20^ In rats it has been shown to both increase^10^ and decrease axonal sprouting.^63^ Stimulation does not decrease the sprouting from TSCs after partial denervation and in fact, may increase it. However, it does decrease bridge formation by these sprouts.^63^

#### Genetic and molecular changes

Transcriptomic and proteomic analysis of nerve injury at the axonal level has identified a pool of genes and their respective products which play a role in both improving and inhibiting axonal regeneration.^85^ Comparatively, research into transcriptomic and proteomic analysis of nerve injured muscle is in its infancy.^86^, ^87^, ^88^ Understanding the differential expressed genes at the level of the neuromuscular junction following nerve injury would allow scientists to investigate the role of their products, potentially unlocking novel therapeutic options.

## Conclusion

The mechanisms of regeneration at the NMJ summarised above provide multiple opportunities for ongoing research in the field of peripheral nerve injury. Whilst interventions to expedite axonal regrowth are in development, the NMJ remains an important component of improving clinical outcomes. Terminal Schwann cells and the constituents of the post synaptic apparatus may be induced to survive for longer, or to be replenished to levels facilitating functional recovery from greater periods of denervation. Stem cell delivery, electrical stimulation and molecular changes are three targets for future research to develop novel methods of maintaining the injured motor end plate.

## Funding

None.

## Declaration of competing interest

None declared.

## Appendix Supplementary materials

Image, application 1

## References

[1] Richter H.P., Peter U. (1982). Impairment of motor function recovery after late nerve suture: experimental study in the rabbit. Neurosurgery.

[2] Reynolds M.L., Woolf C.J. (1992). Terminal Schwann cells elaborate extensive processes following denervation of the motor endplate. J Neurocytol.

[3] Modla S., Mendonca J., Czymmek K.J. (2010). Identification of neuromuscular junctions by correlative confocal and transmission electron microscopy. J Neurosci Methods.

[4] Ann E.S., Mizoguchi A., Okajima S., Ide C. (1994). Motor axon terminal regeneration as studied by protein gene product 9.5 immunohistochemistry in the rat. Arch Histol Cytol.

[5] Gonzenbach H.R., Waser P.G. (1973). Electron microscopic studies of degeneration and regeneration of rat neuromuscular junctions. Brain Res.

[6] Jablonka-Shariff A., Balta E., Santosa K.B., Lu C.Y., Snyder-Warwick A.K. (2023). Terminal Schwann cells are essential for neuromuscular junction function and recovery after nerve injury. Plast Reconstr Surg.

[7] Wang S., Kawabuchi M., Zhou C.J. (2004). The spatiotemporal characterization of endplate reoccupation, with special reference to the superposition patterns of the presynaptic elements and the postsynaptic receptor regions during muscle reinnervation. J Peripher Nerv Syst.

[8] Astrow S.H., Qiang H., Ko C.P. (1998). Perisynaptic Schwann cells at neuromuscular junctions revealed by a novel monoclonal antibody. J Neurocytol.

[9] Saito A., Zacks S. (1969). Fine structure observations of denervation and reinnervation of neuromuscular junctions in mouse foot muscle. J Bone Joint Surg.

[10] Eberstein A., Pachter B.R. (1986). The effect of electrical stimulation on reinnervation of rat muscle: contractile properties and endplate morphometry. Brain Res.

[11] Marques M.J., Pereira E.C.L., Minatel E., Neto H.S. (2006). Nerve-terminal and Schwann-cell response after nerve injury in the absence of nitric oxide. Muscle Nerve.

[12] Tam S.L., Gordon T. (2003). Neuromuscular activity impairs axonal sprouting in partially denervated muscles by inhibiting bridge formation of perisynaptic Schwann cells. J Neurobiol.

[13] Bermedo-García F., Zelada D., Martínez E., Tabares L., Henríquez J.P. (2022). Functional regeneration of the murine neuromuscular synapse relies on long-lasting morphological adaptations. BMC Biol.

[14] Ijkema-Paassen J., Meek M.F., Gramsbergen A. (2002). Reinnervation of muscles after transection of the sciatic nerve in adult rats. Muscle Nerve.

[15] Kosco E.D., Jing H., Chen P. (2023). DOK7 Promotes NMJ regeneration after nerve injury. Mol Neurobiol.

[16] Chen V.Y., Gonzales L.P., Johnston T.R., Steward O., Gupta R. (2023). Preoperative muscle biopsy to assess motor end plate integrity as a predictor for successful nerve transfer. JBJS Case Connect.

[17] Eränkö O., Teräväinen H. (1967). Cholinesterases and eserine-resistant carboxylic esterases in degenerating and regenerating motor end plates of the rat. J Neurochem.

[18] Korneliussen H., Wærhaug O. (1973). Three morphological types of motor nerve terminals in the rat diaphragm, and their possible innervation of different muscle fiber types. Z für Anat Entwicklungsgeschichte.

[19] Kumai Y., Ito T., Matsukawa A., Yumoto E. (2005). Effects of denervation on neuromuscular junctions in the thyroarytenoid muscle. Laryngoscope.

[20] Frank E., Gautvik K., Sommerschild H. (1976). Persistence of junctional acetylcholine receptors following denervation. Cold Spring Harb Symp Quant Biol.

[21] Marques M.J., Conchello J.A., Lichtman J.W. (2000). From plaque to pretzel: fold formation and acetylcholine receptor loss at the developing neuromuscular junction. J Neurosci.

[22] Gupta R., Chan J.P., Uong J. (2020). Human motor endplate remodeling after traumatic nerve injury. J Neurosurg.

[23] Ruven C., Li W., Li H., Wong W.M., Wu W. (2017). Transplantation of embryonic spinal cord derived cells helps to prevent muscle atrophy after peripheral nerve injury. Int J Mol Sci.

[24] Lin H., Xu Z., Liu Y., Chen A., Hou C. (2012). The effect of severing L6 nerve root of the sacral plexus on lower extremity function: an experimental study in rhesus monkeys. Neurosurgery.

[25] Rochel S., Robbins N. (1988). Effect of partial denervation and terminal field expansion on neuromuscular transmitter release and nerve terminal structure. J Neurosci.

[26] Tibúrcio F.C., Goto L.S., Mazzer N. (2023). Neuroregeneration and immune response after neurorrhaphy are improved with the use of heterologous fibrin biopolymer in addition to suture repair alone. Muscle Nerve.

[27] Ijkema-Paassen J., Meek M.F., Gramsbergen A. (2001). Transection of the sciatic nerve and reinnervation in adult rats: muscle and endplate morphology. Equine Vet J.

[28] Li D.D., Deng J., Jin B. (2021). Effects of delayed repair of peripheral nerve injury on the spatial distribution of motor endplates in target muscle. Neural Regen Res.

[29] Ito A., Araya Y., Kawai H., Kuroki H. (2022). Ultrasound stimulation inhibits morphological degeneration of motor endplates in the denervated skeletal muscle of rats. Neurosci Insights.

[30] Vannucci B., Magill C.K., Moore A.M. (2019). What is normal? Neuromuscular junction reinnervation after nerve injury. Muscle Nerve.

[31] Ribariĉ S., Stefanovska A., Brzin M., Kogovŝek M., Kroŝelj P. (1991). Biochemical, morphological, and functional changes during peripheral nerve regeneration. Mol Chem Neuropathol.

[32] Pinto C.G., Tibúrcio F.C., Goto L.S. (2021). Heterologous fibrin biopolymer associated to a single suture stitch enables the return of neuromuscular junction to its mature pattern after peripheral nerve injury. Injury.

[33] Panaite P., Barakat-Walter I. (2010). Thyroid hormone enhances transected axonal regeneration and muscle reinnervation following rat sciatic nerve injury. J Neurosci Res.

[34] Matsuda Y., Ueda K., Sawaizumi T. (1988). Scanning electron microscopic study of denervated and reinnervated neuromuscular junction. Muscle Nerve.

[35] Sakuma M., Gorski G., Sheu S.H. (2016). Lack of motor recovery after prolonged denervation of the neuromuscular junction is not due to regenerative failure. Eur J Neurosci.

[36] Hong S.M., Khang S.K., Kim K.K., Bae Y., Park S.H. (2000). A case of myasthenia gravis proven by ultrastructural study. J Korean Méd Sci.

[37] Fambrough D.M., Hartzell H.C. (1972). Acetylcholine receptors: number and distribution at neuromuscular junctions in rat diaphragm. Science.

[38] Kang H., Tian L., Mikesh M., Lichtman J.W., Thompson W.J. (2014). Terminal Schwann cells participate in neuromuscular synapse remodeling during reinnervation following nerve injury. J Neurosci.

[39] Rich M., Lichtman J. (1989). In vivo visualization of pre- and postsynaptic changes during synapse elimination in reinnervated mouse muscle. J Neurosci.

[40] Mozaffar T., Gupta R., Steward O. (2009). Neuromuscular junction integrity after chronic nerve compression injury. J Orthop Res.

[41] Chan J.P., Gupta R., Uong J. (2020). Examination of the human motor endplate after brachial plexus injury with two-photon microscopy. Muscle Nerve.

[42] Krause M., Wernig A. (1985). The distribution of acetylcholine receptors in the normal and denervated neuromuscular junction of the frog. J Neurocytol.

[43] Li B., Chen L., Gu Y.D. (2020). Stability of motor endplates is greater in the biceps than in the interossei in a rat model of obstetric brachial plexus palsy. Neural Regen Res.

[44] Padovano W., Santosa K.B., Lu C.Y. (2024). 06. Imaging muscle denervation by detecting glutamate carboxypeptidase ii expression with positron emission tomography (PET). Plast Reconstr Surg-Glob Open.

[45] O’Malley J.P., Waran M.T., Balice-Gordon R.J. (1999). In vivo observations of terminal Schwann cells at normal, denervated, and reinnervated mouse neuromuscular junctions. J Neurobiol.

[46] Gorio A., Carmignoto G., Finesso M., Polato P., Nunzi M.G. (1983). Muscle reinnervation—II. Sprouting, synapse formation and repression. Neuroscience.

[47] Lu J.C.Y., Huang Y.H., Chen A.C.Y., Chuang D.C.C. (2023). Using the neuromuscular junction cellular response to predict functional recovery AfterNerve grafting in a mice experimental model. Plast Reconstr Surg.

[48] Ding R. (1982). Lack of correlation between physiological and morphological features of regenerating frog neuromuscular junctions. Brain Res.

[49] Letinsky M.S., Fischbeck K.H., McMahan U.J. (1976). Precision of reinnervation of original postsynaptic sites in frog muscle after a nerve crush. J Neurocytol.

[50] Love F.M., Thompson W.J. (1998). Schwann cells proliferate at rat neuromuscular junctions during development and regeneration. J Neurosci.

[51] Love F.M., Thompson W.J. (1999). Glial cells promote muscle reinnervation by responding to activity-dependent postsynaptic signals. J Neurosci.

[52] Woolf C., Reynolds M.L., Chong M.S. (1992). Denervation of the motor endplate results in the rapid expression by terminal Schwann cells of the growth-associated protein GAP-43. J Neurosci.

[53] Mehta A., Reynolds M.L., Woolf C.J. (1993). Partial denervation of the medial gastrocnemius muscle results in growth-associated protein-43 immunoreactivity in sprouting axons and schwann cells. Neuroscience.

[54] Lu C.Y., Jablonka-Shariff A., Balta E. (2020). Macrophage-derived vascular endothelial growth factor-A is integral to neuromuscular junction reinnervation after nerve injury. J Neurosci.

[55] Magill C.K., Moore A.M., Borschel G.H. (2007). Reinnervation of the tibialis anterior following sciatic nerve crush injury: a confocal microscopic study in transgenic mice. Exp Neurol.

[56] Perez-Gonzalez A.P., Sleigh J.N., Tomlinson R.E. (2022). Functional adaptation of glial cells at neuromuscular junctions in response to injury. Glia.

[57] Dennis M.J., Miledi R. (1974). Non-transmitting neuromuscular junctions during an early stage of end-plate reinnervation. J Physiol.

[58] Slack J.R., WG H. (1982). Neuromuscular transmission at terminals of sprouted mammalian motor neurones. Brain Res.

[59] Bennett M.R., Raftos J. (1977). The formation and regression of synapses during the re-innervation of axolotl striated muscles. J Physiol.

[60] Herrera A., Grinnell A. (1985). Effects of changes in motor unit size on transmitter release at the frog neuromuscular junction. J Neurosci.

[61] Magill C.K., Moore A.M., Borschel G.H., Mackinnon S.E. (2010). A new model for facial nerve research. Arch Facial Plast Surg.

[62] Brown M.C., Ironton R. (1978). Sprouting and regression of neuromuscular synapses in partially denervated mammalian muscles. J Physiol.

[63] Love F.M., Son Y., Thompson W.J. (2003). Activity alters muscle reinnervation and terminal sprouting by reducing the number of schwann cell pathways that grow to link synaptic sites. J Neurobiol.

[64] Thompson W., Jansen J.K.S. (1977). The extent of sprouting of remaining motor units in partly denervated immature and adult rat soleus muscle. Neuroscience.

[65] Zhou C.J., Kawabuchi M., Wang S., Liu W.T., Hirata K. (2002). Age differences in morphological patterns of axonal sprouting and multiple innervation of neuromuscular junctions during muscle reinnervation following nerve crush injury. Ann Anat - Anat Anz.

[66] Bruno C., Cuppini R., Sartini S. (1993). Regeneration of motor nerves in bilobalide-treated rats. Planta Med.

[67] Haimann C., Mallart A., Ferré J.T., Zilber-Gachelin N.F. (1981). Interaction between motor axons from two different nerves reinnervating the pectoral muscle of Xenopus laevis. J Physiol.

[68] Seitz M., Grosheva M., Skouras E. (2011). Poor functional recovery and muscle polyinnervation after facial nerve injury in fibroblast growth factor-2−/− mice can be improved by manual stimulation of denervated vibrissal muscles. Neuroscience.

[69] Favero M., Buffelli M., Cangiano A., Busetto G. (2010). The timing of impulse activity shapes the process of synaptic competition at the neuromuscular junction. Neuroscience.

[70] Gillingwater T.H., Thomson D., Ribchester R.R. (2004). Myo-GDNF increases non-functional polyinnervation of reinnervated mouse muscle. NeuroReport.

[71] Tuffaha S., Lee E.B. (2024). Growth factors to enhance nerve regeneration approaching clinical translation. Hand Clin.

[72] Senger J.L., Power H., Moore A.M. (2024). Electrical stimulation how it works and how to apply it. Hand Clin.

[73] Gordon T. (2024). Physiology of nerve regeneration key factors affecting clinical outcomes. Hand Clin.

[74] Reichenberger M.A., Mueller W., Hartmann J. (2016). ADSCs in a fibrin matrix enhance nerve regeneration after epineural suturing in a rat model. Microsurgery.

[75] Dong S., Zhang Z., Liu H. (2021). Nerve suture combined with ADSCs injection under real-time and dynamic NIR-II fluorescence imaging in peripheral Nerve regeneration in vivo. Front Chem.

[76] Masgutov R., Masgutova G., Mullakhmetova A. (2019). Adipose-derived mesenchymal stem cells applied in fibrin glue stimulate peripheral nerve regeneration. Front Med.

[77] Saller M.M., Prall W.C., Docheva D. (2018). Validation of a novel animal model for sciatic nerve repair with an adipose-derived stem cell loaded fibrin conduit. Neural Regen Res.

[78] Nakada M., Nishibori M., Yano H. (2020). Effects of hybridization of decellularized allogenic nerves with adipose-derive stem cell sheets to facilitate nerve regeneration. Brain Res.

[79] Liu G., Cheng Y., Guo S. (2011). Transplantation of adipose-derived stem cells for peripheral nerve repair. Int J Mol Med.

[80] Durço DFPA, Cardoso G.B.C., de Souza A.F. (2020). Grafts of human adipose-derived stem cells into a biodegradable poly (acid lactic) conduit enhances sciatic nerve regeneration. Brain Res.

[81] Grumbles R.M., Liu Y., Thomas C.K. (2012). Motoneuron replacement for reinnervation of skeletal muscle in adult rats. J Neuropathol Exp Neurol.

[82] Gu S., Shen Y., Wang A. (2010). Application of fetal neural stem cells transplantation in delaying denervated muscle atrophy in rats with peripheral nerve injury. Microsurgery.

[83] Lavasani M., Thompson S.D., Pollett J.B. (2014). Human muscle–derived stem/progenitor cells promote functional murine peripheral nerve regeneration. J Clin Investig.

[84] Hoffman H., Binet F. (1952). Acceleration and retardatton of the process of axon-sprouttng in partially denervated muscles. Aust J Exp Biol Méd Sci.

[85] Wu W., Ruven C., Li H. (2024). Genes in axonal regeneration. Mol Neurobiol.

[86] Weng J., Zhang P., Yin X., Jiang B. (2018). The whole transcriptome involved in denervated muscle atrophy following peripheral nerve injury. Front Mol Neurosci.

[87] Staunton C.A., Johnston A.P.W., Jackson C.J. (2022). Skeletal muscle transcriptomics identifies common pathways in nerve crush injury and ageing. Skelet Muscle.

[88] Carreras D., Chazeau A., Cattin A.L. (2021). Epigenetic changes governing Scn5a expression in denervated skeletal muscle. Int J Mol Sci.

