# Supplementary material for: The fate of the neuromuscular junction in peripheral nerve injury – current understanding and future research aims: A scoping review
Source: JPRAS Open. 2026 May 1;51:312–29. doi: 10.1016/j.jpra.2026.04.016 (PMC13396745; doi:10.1016/j.jpra.2026.04.016)
Supplement: Supplementary file 1 [file mmc1.pdf]

#	**EMBASE Query**
1	motor end plate/ or nerve ending/ or neuromuscular junction/
2	nerve degeneration/ or degeneration/ or nerve fiber degeneration/ or retrograde degeneration/ or transneuronal degeneration/ or wallerian degeneration/
3	denervation/ or muscle denervation/
4	cell degeneration/ or nerve cell degeneration/ or nerve fiber degeneration/
5	2 or 3 or 4
6	1 and 5
7	(motor endplate* or motor nerve end plate* or motor nerve endplate* or motor end plate* or motor nerve ending* or motor nerve terminal* or motor plate* or muscle end plate* or muscle endplate* or neuromuscular end plate* or neuromuscular endplate* or neuromuscular junction* or neuro muscular junction* or myoneural junction* or myoneural junction* or myoneural synapse? or neuromuscular conduction? or neuromuscular connexion? or neuromuscular contact? or neuromuscular synapse? or neuromyal junction? or motor neuronal terminal or motor nerve end organ).mp.
8	(degenerat* or denervat* or deteriorat* or degradation or damage* or dysfunction*).mp.
9	7 and 8
10	6 or 9
11	peripheral nerve injury/
12	(peripheral adj3 (nerve* or neural) adj5 (injur* or trauma* or crush* or damage* or laceration* or transect* or wound* or lesion*)).mp.
13	cell regeneration/ or cell renewal/ or nerve fiber regeneration/
14	(nerve fiber regeneration or nerve regeneration or axon regeneration or axonal regeneration).mp.
15	((nerve or neural or axon or axonal) adj3 (renewal or revitalization or rejuvenation)).mp.
16	reinnervation.mp.
17	11 or 12 or 13 or 14 or 15 or 16
18	10 and 17

#	**MEDLINE Query**
1	Motor Endplate/ or Nerve Endings/ or Neuromuscular Junction/
2	Nerve degeneration/
3	Denervation/ or Muscle Denervation/
4	Nerve Degeneration/
5	2 or 3 or 4
6	1 and 5
7	(motor endplate* or motor nerve end plate* or motor nerve endplate* or motor end plate* or motor nerve ending* or motor nerve terminal* or motor plate* or muscle end plate* or muscle endplate* or neuromuscular end plate* or neuromuscular endplate* or neuromuscular junction* or neuro muscular junction* or myoneural junction* or myoneural junction* or myoneural synapse? or neuromuscular conduction? or neuromuscular connexion? or neuromuscular contact? or neuromuscular synapse? or neuromyal junction? or motor neuronal terminal or motor nerve end organ).mp.
8	(degenerat* or denervat* or deteriorat* or degradation or damage* or dysfunction*).mp.
9	7 and 8
10	6 or 9
11	peripheral nerve injuries/
12	(peripheral adj3 (nerve* or neural) adj5 (injur* or trauma* or crush* or damage* or laceration* or transect* or wound* or lesion*)).mp.
13	Nerve Regeneration/
14	(nerve fiber regeneration or nerve regeneration or axon regeneration or axonal regeneration).mp.
15	((nerve or neural or axon or axonal) adj3 (renewal or revitalization or rejuvenation)).mp.
16	reinnervation.mp.
17	11 or 12 or 13 or 14 or 15 or 16
18	10 and 17

#	PubMed Query
1	Motor Endplate* or Nerve Ending* or Neuromuscular Junction*
2	Nerve degeneration*
3	Denervation* or Muscle Denervation*
4	Nerve Degeneration*
5	2 or 3 or 4
6	1 and 5
7	motor endplate* or motor nerve end plate* or motor nerve endplate* or motor end plate* or motor nerve ending* or motor nerve terminal* or motor plate* or muscle end plate* or muscle endplate* or neuromuscular end plate* or neuromuscular endplate* or neuromuscular junction* or neuro muscular junction* or myoneural junction* or myoneural junction* or myoneural synapse? or neuromuscular conduction? or neuromuscular connexion? or neuromuscular contact? or neuromuscular synapse? or neuromyal junction? or motor neuronal terminal or motor nerve end organ
8	degenerat* or denervat* or deteriorat* or degradation or damage* or dysfunction*
9	7 and 8
10	6 or 9
11	peripheral nerve injur*
12	peripheral nerve injur* or peripheral nerve trauma* or peripheral nerve crush or peripheral nerve damage* or peripheral nerve laceration* or peripheral nerve transect* or peripheral nerve wound* or peripheral nerve lesion*
13	Nerve Regeneration/
14	nerve fiber regeneration or nerve regeneration or axon regeneration or axonal regeneratio
15	reinnervation.mp.
16	11 or 12 or 13 or 14 or 15
17	10 and 17

#	Web of Science Query
1	Motor Endplate* or Nerve Ending* or Neuromuscular Junction*
2	Nerve degeneration*
3	Denervation* or Muscle Denervation*
4	Nerve Degeneration*
5	2 or 3 or 4
6	1 and 5
7	motor endplate* or motor nerve end plate* or motor nerve endplate* or motor end plate* or motor nerve ending* or motor nerve terminal* or motor plate* or muscle end plate* or muscle endplate* or neuromuscular end plate* or neuromuscular endplate* or neuromuscular junction* or neuro muscular junction* or myoneural junction* or myoneural junction* or myoneural synapse? or neuromuscular conduction? or neuromuscular connexion? or neuromuscular contact? or neuromuscular synapse? or neuromyal junction? or motor neuronal terminal or motor nerve end organ
8	degenerat* or denervat* or deteriorat* or degradation or damage* or dysfunction*
9	7 and 8
10	6 or 9
11	peripheral nerve injur*
12	peripheral nerve injur* or peripheral nerve trauma* or peripheral nerve crush or peripheral nerve damage* or peripheral nerve laceration* or peripheral nerve transect* or peripheral nerve wound* or peripheral nerve lesion*
13	Nerve Regeneration/
14	nerve fiber regeneration or nerve regeneration or axon regeneration or axonal regeneratio
15	reinnervation.mp.
16	11 or 12 or 13 or 14 or 15
17	10 and 17

Additional information:

No language restrictions
No time restrictions
Grey literature will also be searched
References of included studies will also be reviewed
